# Effect of the Agglomerate Geometry on the Effective Electrical Conductivity of a Porous Electrode

**DOI:** 10.3390/membranes11050357

**Published:** 2021-05-14

**Authors:** Abimael Rodriguez, Roger Pool, Jaime Ortegon, Beatriz Escobar, Romeli Barbosa

**Affiliations:** 1 División de Ciencias e Ingeniería, Universidad de Quintana Roo, Boulevard Bahía s/n, Chetumal 77019, Quintana Roo, Mexico; abima777@gmail.com (A.R.); rogerrpcc@gmail.com (R.P.); jortegon@uqroo.edu.mx (J.O.); 2 Unidad de Energía Renovable, Centro de Investigación Científica de Yucatán, C 43 No 130, Chuburná de Hidalgo, Mérida 97200, Yucatán, Mexico; bem08@hotmail.com

**Keywords:** effective transport coefficients, percolation, polygonal synthetic images, statistical descriptors

## Abstract

The study of the microstructure of random heterogeneous materials, related to an electrochemical device, is relevant because their effective macroscopic properties, e.g., electrical or proton conductivity, are a function of their effective transport coefficients (ETC). The magnitude of ETC depends on the distribution and properties of the material phase. In this work, an algorithm is developed to generate stochastic two-phase (binary) image configurations with multiple geometries and polydispersed particle sizes. The recognizable geometry in the images is represented by the white phase dispersed and characterized by statistical descriptors (two-point and line-path correlation functions). Percolation is obtained for the geometries by identifying an infinite cluster to guarantee the connection between the edges of the microstructures. Finally, the finite volume method is used to determine the ETC. Agglomerate phase results show that the geometry with the highest local current distribution is the triangular geometry. In the matrix phase, the most significant results are obtained by circular geometry, while the lowest is obtained by the 3-sided polygon. The proposed methodology allows to establish criteria based on percolation and surface fraction to assure effective electrical conduction according to their geometric distribution; results provide an insight for the microstructure development with high projection to be used to improve the electrode of a Membrane Electrode Assembly (MEA).

## 1. Introduction

Due to its fluctuating and intermittent nature, the storage of renewable energy is a challenge. Therefore, hydrogen (H2) is projected as an energy vector and can be used by fuel cells (FC) [1,2]. FC are electrochemical devices that continuously and directly convert the chemical energy of a fuel into electrical energy [3]. The FC are classified according to the type of electrolyte they use, being considered the proton exchange membrane fuel cell (PEMFC) among the most promising [4,5]. Because hydrogen is not found as a free element naturally, it is necessary to produce it. The process for hydrogen production and storage demands large amounts of energy, so increasing the performance of PEMFCs translates into better use of the hydrogen produced. The catalytic layer (CL) of the PEMFCs is the component responsible for carrying out the transformation of chemical to electrical energy [6,7] and it is part of the so-called random heterogeneous materials (RHM). RHM are used in various engineering applications such as batteries, supercapacitors, and membrane electrode assemblies (MEA) of PEMFC’s [8]. From RHM, different types of arrangement of two or more phases can be distinguished at the microstructural level, in which phenomena of mass and energy transport can occur, resulting in a valuable effect such as an electric charge based on its effective transport coefficients (ETC). There are several works about calculating the material conduction efficiency from real images taken of materials as well as synthetic images to predict the behavior of PEMFCs [9,10]. The significant advance in the representation of material models and their microstructural properties still require improvements, mainly for the use and prediction of real three-dimensional models. A proposal that has been widely accepted is the representation of models through synthetic images, mainly in the improvement of microstructural behavior of various types of materials. Among the applications using synthetic images are the development of renewable energy such as synthesis of materials and prediction of behaviors for fuel cells [9], devices and apps for medicine (magnetic resonance imaging) [11], neural networks mainly with the use of Deep Learning [12], materials for ultra-fast devices in the telecommunications area (ultra-fast devices) [13,14], military applications such as radars and ship detection simulators [15], and topographical images of polymer solar cells [16]. There are other works involved in the improvement of microstructures related to comparison of different morphologies on 3D reconstructions [17], the behavior of their geometry to conversion of triangular to hexagonal models [18], synthesis of palladium nanoparticles in triangular form [19], Finite Volume Method (FVM) for morphology studies of microstructures with mechanoluminescent particles [20], heat and humidity transfer in clothing sets, using the finite volume method for the nonlinear parabolic equations system [21], computational thermal conductivity and membrane pore geometry simulation in porous materials [22,23], tortuosity, permeability and threshold percolation studies from membrane SEM images and transport pore structure [24,25,26], images generation from mathematical descriptors for 3D shapes analysis using formal segmentation [27], structural detail analysis of woven fabric based on synthetic images [28], thermal expansion coefficients calculation for one and two phases from SEM models and three-dimensional synthetic images of polycrystals [29], geometric and topological characterizations to establish a relationship of the structure owned by two phases using the Voronoi diagram in geometry of synthetic images [30,31], neutron imaging in fuel cells research [32], and a systematic classification implemented by its geometric and topological properties focus on imitating morphology through mathematical tools, such as digital image correlation, tessellation, random field generation, and differential equation solvers [33]. Finally, synthetic anisotropic training is performed to reconstruct anisotropic media [34] and multiscale model-based on synthetic structures, using isotropic filtering [35]. Particularly for PEMFCs, the study of the microstructure of the catalytic layer (CL) and the gas diffuser layer (GDL) are a constant subject of study that aims to improve the performance of fuel cells since its manufacture. Numerical models are usually simplified, transferring from the microstructure domain to a discrete (computational) environment, so the microstructure is represented by images (pixels). However, there is a lack of investigations examining the geometry influence in conduction transport problems. For this reason, numerical analyses are implemented using synthetic images to determine the behavior of different polygonal configurations and their repercussions on the effective electrical conductivity, considering percolation and tortuosity parameters. This approach can provide a new insight in achieving high conduction values which can be applied to scanning electron microscope images.

## 2. Materials and Methods

**Figure 1:**
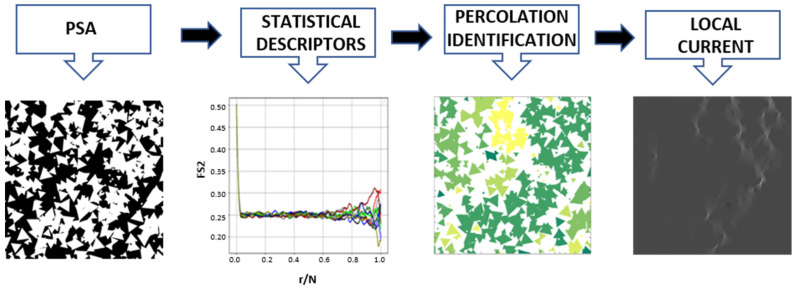
Methodology scheme.

In the last years, the study of different multiform geometry by continuous mathematics and numerical approximation has been increased in the computer graphics area [16,17,18,19,20,21,22,23,24,25,26,27]. In this work, an algorithm is developed to analyze the geometric behavior of polygonal synthetic agglomerate (PSA) from circles and polygons of 3–5 sides. Figure 1 presents the methodology developed in this work in four stages. The first step is to generate the geometric structure modeling (two-dimensional PSA) from mathematical descriptors. PSA needs to be statistically characterized by two-point and linear path correlation functions. Subsequently, percolation is obtained through structure identification modeling of an infinite cluster. Finally, FVM is used to determine the effective transport coefficients and local current from PSA. This method considers each phase as conductive and non-conductive, respectively.

### 2.1. PSA Generation Process

**Figure 2:**
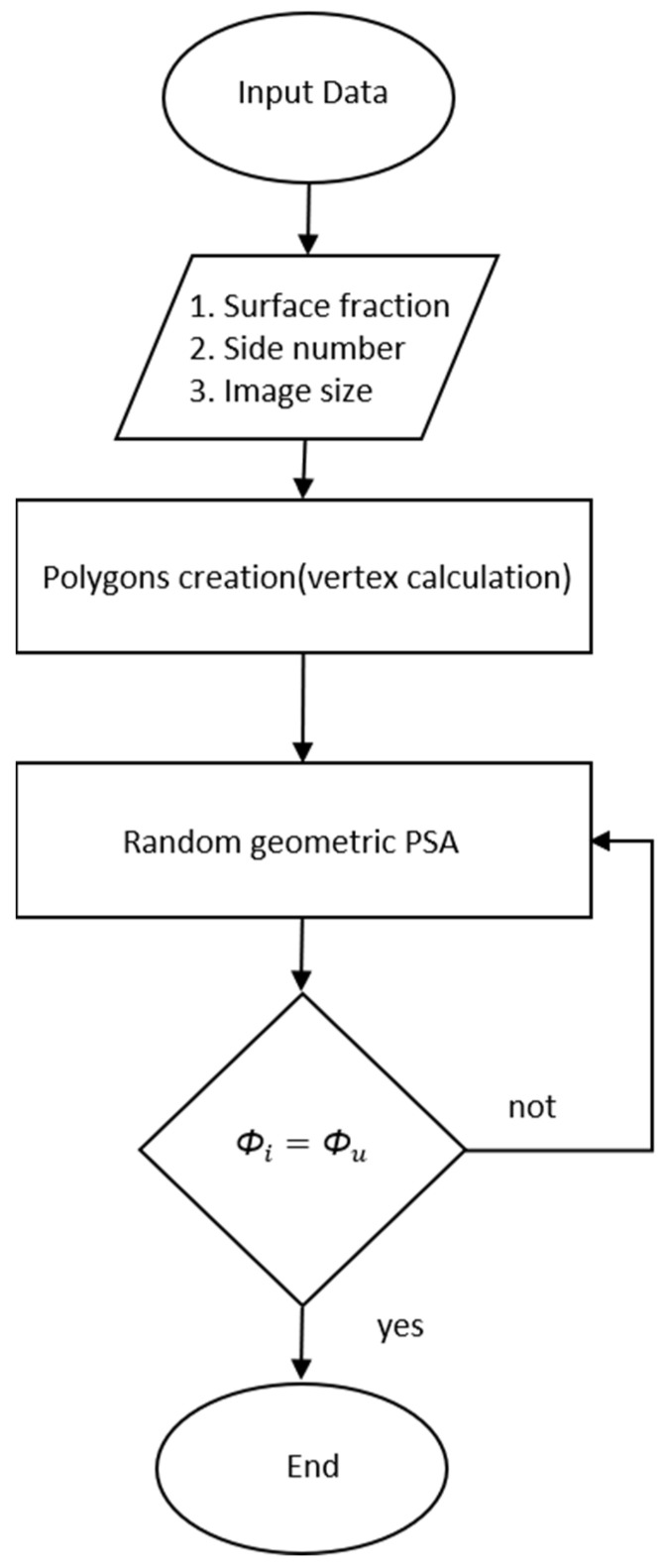
Flowchart for PSA generation.

The PSA generation process is carried out through a series of steps described in Figure 2.

First, initial parameters are necessary to generate the two-phase synthetic image, which is defined considering the size of the matrix, geometry, and surface fraction of PSA. The size is defined as *m* × *m*, where *m* is the number of pixels per row and column. Zero and one values correspond to the black and the white pixels which are inserted in a matrix, respectively. The surface fraction is the ratio between ones and zeros in the matrix. There are different techniques for the generation of synthetic images based on mathematical descriptors [27,28,29,30,31,32,33]. The technique used in this work is based on the union of points called vertices, for the formation of the PSA. PSA are created from circles and polygons with three, four, and five sides called generator figures. In the case of circles, the circumference equation is used. Another smaller defined matrix contains the generating figures.

**Figure 3:**
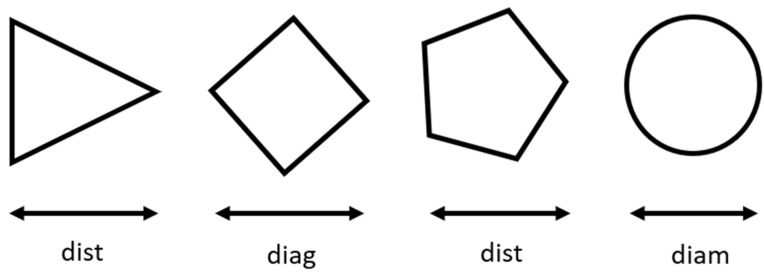
Different Geometry of PSA generation.

Figure 3 shows PSA generation for different geometries such as circles and 3, 4, 5-sided polygons where dist is the distance between the left base and the right vertex for 3 and 5 sided polygons, diag is the diagonal size for 4-sided polygon, and diam is the diameter for all circles in pixels.

The angles that correspond to each vertex to determine the points that form the polygons can be calculated by Equation (1):

(1) $$\theta (e)=e\cdot \frac{360{}^{\circ}}{L}$$

where *θ* is the angle of the vertex position relative to the center, *L* is the number of sides of the polygon and *e* is the vertex index.

**Figure 4:**
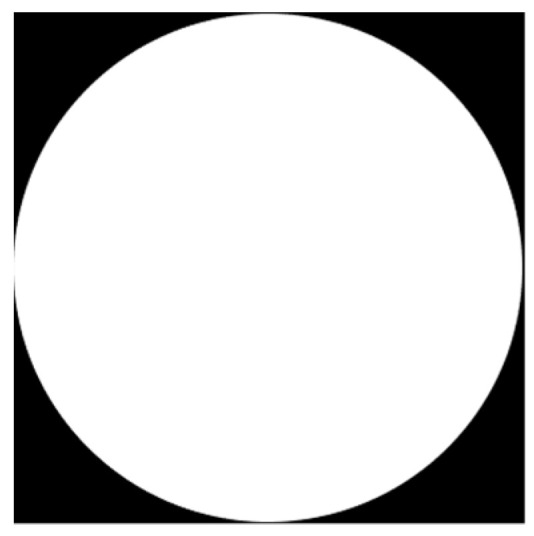
Example of image generation for circle PSA.

Figure 4 shows an example of geometry generation. The technique used to insert the generating image takes the size of the structural element which will then be captured in the matrix, centered on a given matrix point, following a model like [36] but applied to the linear representation of the polygon contour trough of vertices. To implement a PSA, the determination of pixels is made from the number of vertices (depending on the geometry) of a region that are only partially covered by the borderline. Pixels may be partially covered by the edge of a region of interest. To determine which pixels are in the region, a sub-grid is used considering pixels that are inside the polygon. Each time the PSA is updated with a new insertion, the surface fraction is calculated until a threshold value is reached.

**Figure 5:**
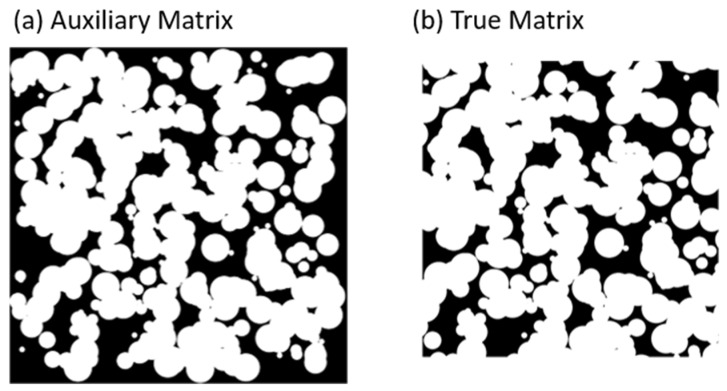
Example of the full process for circle Matrices shape resulting from applying initial parameters and geometries. (**a**) shows AuxiliaryMatrix with zeros and ones assigned and (**b**) shows the composition of the resulting matrix (TrueMatrix) from the image cropping of the auxiliary matrix.

Figure 5 shows a comparison between an original matrix (Figure 5a) and cropped matrix (Figure 5b) for PSA circle generation. The Auxiliary Matrix is a binary image that outlines the definitive region boundaries of the Matrix composition (Figure 5a), obtained saturating non-zero pixels. A stochastic morphology is generated because the initialization is random.

**Figure 6:**
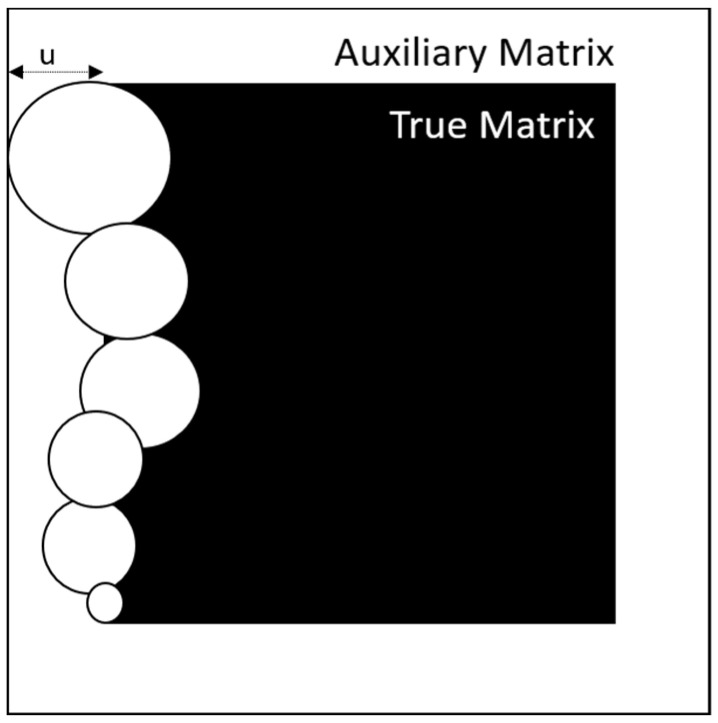
Identification process at the edge for the image cropping (TrueMatrix) from Auxiliary matrix. From each of the sides of the matrix, it is removed to obtain a TrueMatrix with the size of the desired matrix where u is the maximum diameter divided by 2.

The technique used for image cropping is shown in Figure 6 where AuxiliaryMatrix is larger by the maximum size of a complete generating figure towards the four cardinal points. The designed algorithm requires cropping the image to remove the unwanted frame; the final matrix, TrueMatrix, has the desired image size (Figure 5b).

### 2.2. Statistical Descriptors

Because of their microstructural complexity, RHMs are challenging to characterize, but statistically, they can yield characteristics that cannot be deduced with standard analysis methods. Several parameters, such as volumetric fractions of the phases, quantification of the surface area, orientation, size distributions, phase connectivity, among others, have been used to describe RHMs in detail. Statistical descriptors are the point correlation functions that have been used to describe microstructures statistically [9]. Correlation functions are based on the idea that a complex porous structure can be described by the values of a phase function, within the porous medium. The phase function takes the value of zero or one, depending on where the point is located, and it can be defined according to the following Equation (2) [37]:

(2) $$T_{\pi }(x)=\{\begin{array}{c} 1,\;if\;x\;\in \;\pi \\ 0,\;otherwise \end{array}$$

According to Equation (3), the surface fraction of the phase ***π*** is defined as the average of the phase function (*x*).

(3) $$\phi_{\pi }=\langle T_{\pi }(x)\rangle$$

#### 2.2.1. Two-Point Correlation Function

A correlation function can extract statistical information from a dimensional subspace of a moderate size. The two-point correlation function is an important statistical parameter for the description of isotropic RHM, which indicates the probability that two points separated by a linear distance coincide in the same phase. The unit of measurement of the points for this case is a pixel. For an isotropic RHM, this function can be obtained by randomly throwing a line segment of length r with specific orientation and counting the number of times that the start (*x*) and end (*x + r*) of the line are in the phase. The two-point correlation function is defined by Equation (4) [38]:

(4) $$S_{2,\pi }(x,r)=\langle T(x)\;T(x+r)\rangle$$

where *x* denotes the position of an arbitrary point within the computational domain, π=0, 1, 2, …, n is the phase of the porous medium, and r is the distance from $x_{1}$ to $x_{2}$**.** Two-point correlation function is a great statistical descriptor, and due to the simplicity in its application in computer programs, it will be used as an indicator of connectivity between phases.

From $S_{2,\pi }(x,r)$, we can also define the autocovariance function,

(5) $$\chi_{\pi }(x,r)=S_{2,\pi }(x,r)-\phi_{\pi }^{2}$$

and its normalized function,

(6) $$\chi_{\pi }^{*}=\frac{\chi_{\pi }(x,r)}{\phi_{\pi }(1-\phi_{\pi })}$$

#### 2.2.2. Line-Path Correlation Function

The line-path correlation function provides statistical information about the conductivity of the sample phases, being this the probability that a segment of points (each one separated by a discrete space) belongs to a straight line. From the previous phase definition, the conductivity is validated if all the pixels that make up the line belong to the same phase. In an isotropic RHM, the line-path correlation function only depends on the length of the *r* line. When *r* = 0 the line-path correlation function is equal to the surface fraction of the studied phase. Equation (7) shows the line-path correlation function mathematical form defined as:

(7) $$L_{p,\pi }(x,r)=\langle \sum_{0}^{r}T_{\pi }(x+i)\rangle$$

The normalized line-path correlation function can be defined by the following equation [38],

(8) $$L_{\pi }^{*}=\frac{L_{p,\pi }(x,r)}{\phi_{\pi }}$$

#### 2.2.3. Average Correlation Function

In this work, the average correlation function is used as statistical characterization, which includes the averaged values of the normalized correlation functions obtained in Equations (6) and (8) and determined for the Ω(ω) ensemble:

(9) $$F_{\left(\Omega ,r\right)}=\sum_{\omega =1}^{10}(\frac{\chi_{\pi }^{*}+L_{\pi }^{*}}{2})/10$$

where ω is the number of configurations.

### 2.3. Conduction Efficiency

The effective properties of the RHMs are functions of the individual properties, volumetric fractions, and the microstructural design. The conduction efficiency is calculated from the iterative FVM and provides information about the properties of the material. The ETC of RHM can be defined as the proportionality coefficient that characterizes the material’s entire domain. Then, ETC value is essential to know the behavior of conduction for designing devices. For RHM consisting of *n* phases, the general function $K_{e}$ is described according to Equation (10):

(10) $$K_{e}=f(K_{1},\;K_{2},\;\ldots ,\;K_{n};\;\Phi_{1},\;\Phi_{2},\;\ldots \;\Phi_{n};\;\Omega )$$

where $K_{e}$. is the effective conductivity, *K_i_* is the proportionality constant for that phase, $\phi_{i}$ is the composition of the surface fraction and *Ω* is the structure of the phases. For energy applications, relevant ETCs are thermal conductivity, electrical conductivity, dielectric constant, magnetic permeability, and diffusion coefficient [6]. It is important to emphasize that the microstructural information from RHM is generally not a simple relationship. The ETCs in a discrete (computational) environment can be calculated employing the local fields, which must be derived from the appropriate theory according to the problem in question [39]. In problems where conduction is relevant, the effective properties are defined by a linear relationship between the averages of both a generalized local flow *J* and an applied potential *E* [38], as indicated by Equation (11):

(11) $$J\propto K_{e}\cdot E\;$$

For charge conduction problems, the average generalized flux (*J*) represents the average local electric current and the applied average potential (*E*) represents the electric field. For electric current we have Ohm’s law given by Equation (12):

(12) $$I=\frac{1}{R}\Delta E$$

where *I* is the electric intensity, *R* is the electrical resistance and *E* is the electric potential difference. Considering an RHM, $K_{e}$. can be calculated through conductance, where *k* is related to conductivity, *A* is the transversal area, and *L* is the charge transport length, relative to the flow direction given by Equation (13).

(13) $$K_{e}=\frac{kA}{L}$$

$J_{eff}$ is an effective value for the RHM determined by Equation (14).

(14) $$J_{eff}=K_{e}\cdot \Delta E$$

Now, it is necessary to introduce the concept of conduction efficiency $\varepsilon_{k}$, which is derived directly from the second law of thermodynamics. The conduction efficiency ($\varepsilon_{k}$) is calculated from Equation (15), which provides the relationship to obtain the effectiveness in the catalytic layer described in [6].

(15) $$\varepsilon_{k}=100\cdot \frac{K_{e}}{K_{M}}$$

where $K_{M}$ is the nominal conductivity.

### 2.4. Percolation

The percolation theory is carried out under two approaches. Physical percolation theory deals with phenomena such as the electric current conduction, thermoelectric phenomena, elastic, and non-elastic deformations in diverse media, among others; meanwhile, the geometric percolation theory deals with phenomena that are responsible for the analysis of microstructure connection of different phases and connections between boundaries [40]. Considering a two-dimensional system, each site in the mesh of this system can be occupied randomly and independently with a probability p, where the sites with at least one side in common are known as the closest neighbors. A cluster is a group of neighboring sites. The sites connected directly will be called connections (sites occupied with agglomerate phase), and the rest of the connections will be called no connections (sites occupied with matrix phase). The border connections which connect from border to border are known as infinite clusters [41]. If an infinite cluster is confirmed, the existence of the percolating phenomenon is assured. In an infinite cluster can be recognized several sections where the current flows smoothly.

The physical properties, that involve the transport phenomena, present the percolation problem [42], where the percolation threshold is a pore density number that varies its size from smaller to larger. It varies in a ratio directly proportional to the number of pore densities. The percolation process and the electrical conductivity can be related if the problem is represented with a microstructure with random connections where the agglomerate phase has a p number of connections and 1-p corresponds to connections with the matrix phase. In an agglomerate phase (conductive material), the number of connections is related to the pore density number. The larger the identified clusters of a phase in a heterogeneous material, the more influence there will be on its microstructural properties [41].

## 3. Results and Discussions

Results are presented using a *Ω* ensemble of ten different random series (*W* = 10) for four different PSA from random mathematical descriptors with its surface fraction controlled. The main algorithm was implemented in C++; an Alienware Aurora with Intel Core I7-870 and 64 Gb was used for concurrent executions.

### 3.1. PSA Generation Process

**Figure 7:**
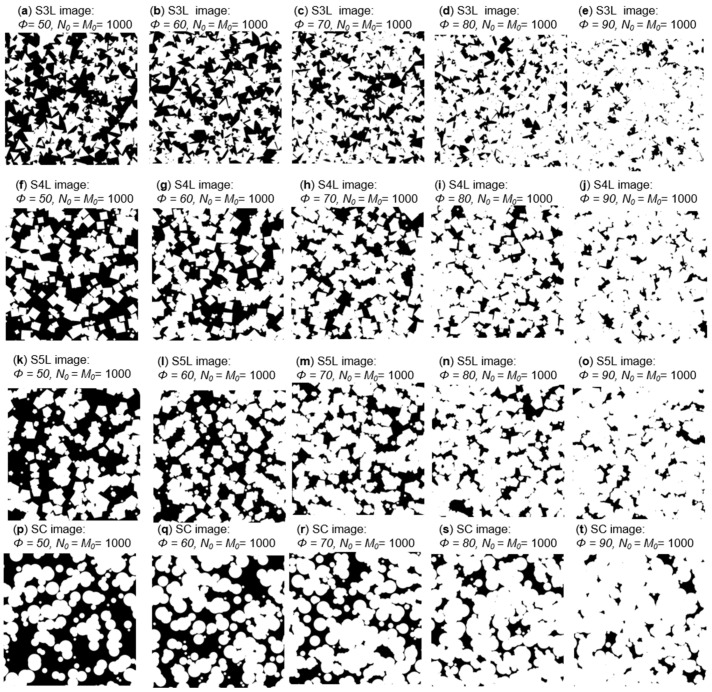
Constructed synthetic media for different geometry of agglomerate phase in a surface fraction range of 50% to 90%. (**a**–**e**) is for S3L image, (**f**–**j**) is for S4L image, (**k**–**o**) is for S5L image and (**p**–**t**) is for SC image.

A total of 360 PSA with four configurations were generated for experimentation: 90 PSA for each kind of figure (SC for circular geometry, S3L for 3 sides, S4L for 4 sides, and S5L for 5 sides). The surface fraction ($\phi_{j}$), in an interval from 10% to 90% with steps of 10% is considered. The size of each PSA is 1000 × 1000 pixels. Diag, dist, and diam distance for each of the geometries presented is random in the range from 10 up to 100. Figure 7 shows a PSA of the materials studied (S3L, S4L, S5L, and SC) of agglomerate phase with surface fraction in a range of 50% to 90% for every configuration; all different random series follow the same generation process and they only differ in distribution and geometry size.

### 3.2. Statistical Analysis of Microstructures

**Figure 8:**
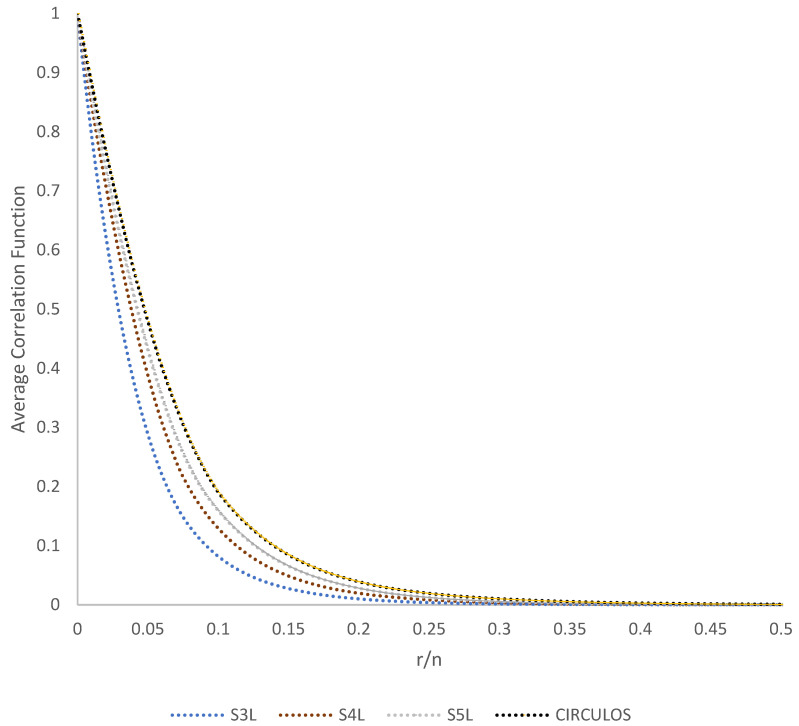
Average correlation functions normalized for a ten PSA realization for every configuration (S3L, S4L, S5L, and SC) generated along the process presented in Figure 4 in a surface fraction range of 10% to 90% for the agglomerate phase.

Figure 8 depicts corresponding results for statistical descriptors for four different PSA geometries with surface fraction controlled. The average correlation functions were taken from the ten PSA of each configuration generated (averaged and normalized) for the indicated surface fractions (Equation (9)). A curve is presented for each of the configurations (S3L, S4L, S5L, and SC). Every case shows periodicity and reveals a monotonic decay to its asymptotic value, which does not guarantee that there is a correlation in spatial elements, mainly because it is the result of an average on each of the configurations. However, it is considered the fact that there may be a statistically significant number of clusters in the system that can better capture the grouping information. It can be seen how S3L decays faster concerning the trend shown by the other correlation functions images, with SC taking the longest time to adjust the curve.

### 3.3. Percolation Process

**Figure 9:**
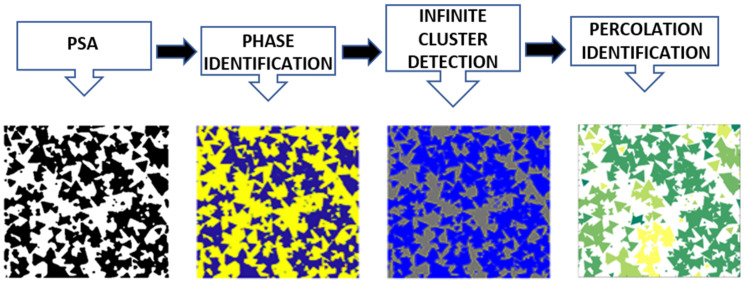
Percolation process for infinite cluster detections.

Low-order correlation functions do not reflect grouping information. For this reason, it is required to know the tendency to group by percolation. The percolation process can be calculated by evaluating the connection of both ends, providing the tendency of every cluster formed to identify an infinite cluster. The full process is shown in Figure 9.

**Figure 10:**
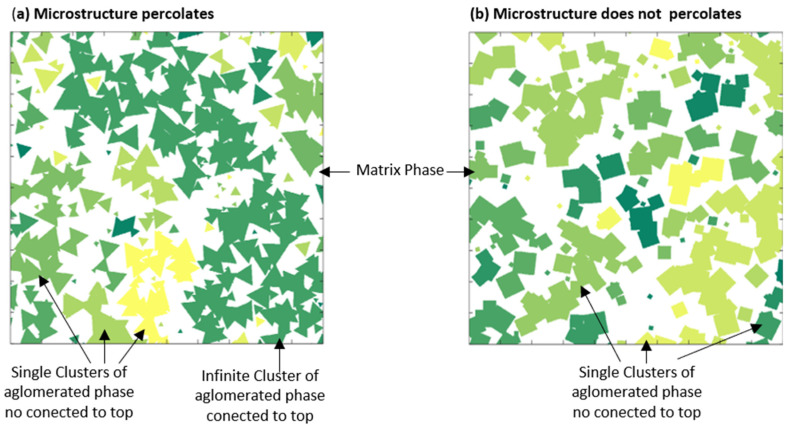
Infinite cluster classification. (**a**) shows different single cluster and an example of infinite cluster when agglomerate phase is connected to top. (**b**) shows single clusters without percolation.

In the first stage, the original PSA obtained from mathematical descriptors is shown. After obtaining the PSA (first step of Figure 9), a process begins to identify how many pixels are connected; these are classified in groups, called clusters, of the same phase (matrix phase or agglomerate phase in the second step of Figure 9). Once the existence of clusters is categorized, the color labeling identification is assigned according to the number of pixels identified. Finally, in the last stage, cluster existence is verified, mainly infinite clusters having a connection between the ends of the interfaces in the microstructure [43]. The percolation process can be better appreciated in Figure 10, which shows the grouping of the clusters of a PSA for two different cases. Figure 10a shows the S3L geometry (white agglomerate phase) in which the different cluster connections are identified according to the pixels. For this reason, Figure 8 percolate. Figure 10b shows the S4L-PSA (white agglomerate phase) with several single clusters are observed, but no infinity cluster (microstructure does not percolate).

**Figure 11:**
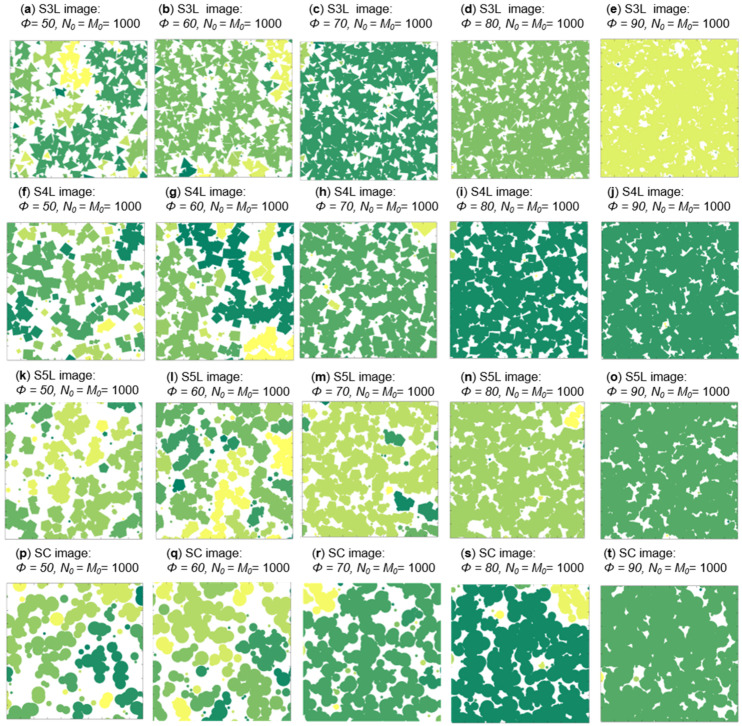
Percolation evaluation for the agglomerate phase of Figure 7 in a surface fraction range of 50% to 90%. (**a**–**e**) show percolation results for S3L images, (**f**–**j**) show percolation results for S4Limages, (**k**–**o**) show percolation results for S5L and (**p**–**t**) show percolation results for SC images.

Figure 11 shows a cluster classification for the agglomerate phase for each configuration. A cluster can be identified by a different color labeling according to the pixel connection found in the same phase cluster. The matrix phase is represented by white color

Table 1 shows the percolation for matrix and agglomerate phases, identifying with 1 when there is percolation in 100% of realizations and zero with no percolation, which is presented as a function of the surface fraction for each configuration in a surface fraction range of 10% to 90%. In the agglomerate phase, the PSA with the best percolation is in the range from 50% to 90% for the S3L while S4L, S5L, and SC present percolation in a range from 70% to 90%.

In the matrix phase, the best performance is in the range from 10% to 60% for SC, while S4L, S5L, and S3L present percolation in a range from 10% to 50%.

### 3.4. Conduction Efficiency

Conduction efficiency and effective local current are calculated from equation 15 for the entire PSA bank considering every realization for both phases through FVM, taking into consideration a classical discretization to solve transport problems and stability analysis. The electric potential in the geometric limits has been implemented as a boundary condition, to have a generalized potential differential (P0–P1). It can be appreciated in [9], the calculation of the effective transport coefficients is performed from reconstructed images from binarized SEM images. On this occasion, PSA images are used to obtain the ETC using FVM with the tridiagonal matrix solution.

**Figure 12:**
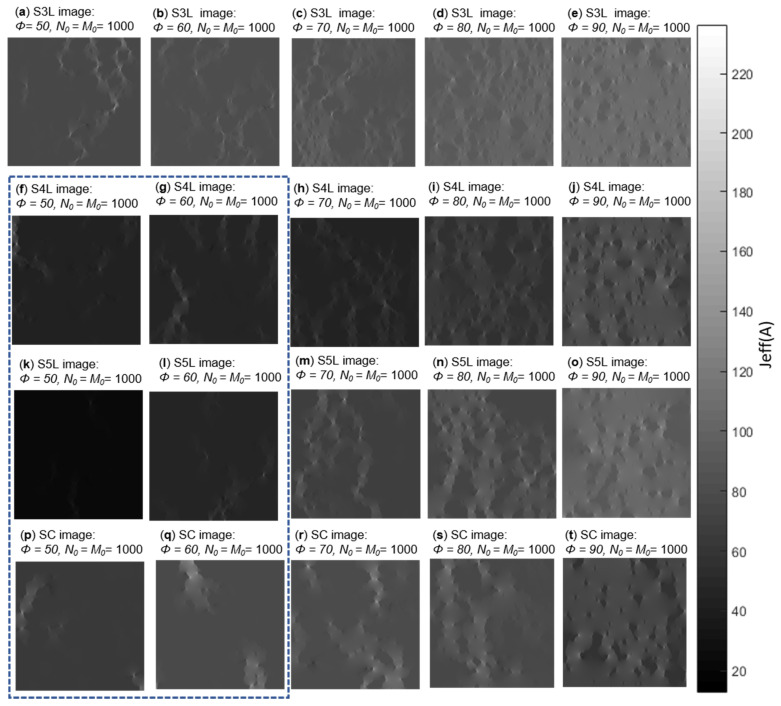
Local current distribution $J_{eff}$ for the agglomerate phase of Figure 7 in a surface fraction range from 50% to 90%. (**a**–**e**) show local current distribution results for S3L images, (**f**–**j**) show local current distribution results for S4L images, (**k**–**o**) show local current distribution results for S5L images and (**p**–**t**) show local current distribution results for SC images. Images en-closed by the dotted line do not have a connection between pixels or cur-rent distribution between their edges.

Figure 12 shows the numerical solution of local current distribution for all disperse phase PSA configurations in a surface fraction range from 50% to 90%. According to local current efficiency and percolation analysis (Table 1), images enclosed by the dotted line (Figure 12f–q) do not have a connection between pixels or current distribution between their edges. S3L current results (Figure 12a–e) is the only configuration that has conduction between its ends in each surface fraction.

Connectivity analysis between the phases can avoid wasting processing time in ETC calculation, dispersion, and variance of data from PSA simulated. Execution time per PSA is 9 h. The runtime for the generation of each PSA and the characterization of the statistical descriptors (two-point correlation function and linear path correlation functions) is 1 h. Once the PSAs are generated, the calculation of the effective transport coefficients is performed in a second module, which takes 8 h per image. Every module is executed concurrently, similar applies to every PSA. When considering 360 realizations, it will be worthwhile to select those PSAs that may have a connection in their interfaces to guarantee ETC results.

The distribution of current can be observed from end to end where some infinite cluster is found, in the range of interest, showing the best performance.

**Figure 13:**
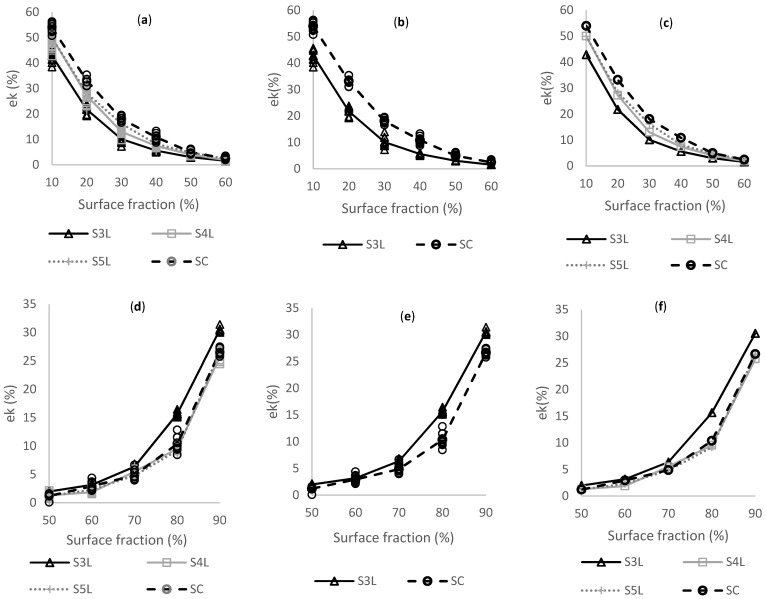
Conduction efficiency ($\varepsilon_{k}$) as a function of ϕ for ω. (**a**–**c**) show conduction efficiency for the matrix phase in a range of 10% to 60% and (**d**–**f**) is for an agglomerate phase in a range of 50% to 90%.

Figure 13 provides a general trend of $\varepsilon_{k}$ (%). Figure 13a–c reveals the behavior of conduction efficiency for the matrix phase in a surface fraction range of 10% to 60%.

Figure 13a shows the averages (continuous line) and ten realizations (markers) per configuration. Figure 13b displays a comparison of the averages and realizations for SC and S3L configuration, and Figure 13c shows only the average values for every configuration. SC configuration has the highest conductivity for the matrix phase while S3L obtains the lowest conduction values for the phase. The lower the surface fraction values, the higher the conductivity in the matrix phase. Figure 13d–f are related to the agglomerate phase in a surface fraction in a scope of 50% to 90%. Figure 13e compares the best conduction efficiency against the worst. Under this premise, the S3L image is the only one that percolates at 50% of surface fraction for the agglomerate phase, presenting for each surface fraction a higher conduction efficiency, while for the matrix phase, it has the lowest levels of conduction. When there is more conduction in one phase, the other one decreases. In the value of fraction 0.5, the behavior of conductivity is the same for both phases. For all configurations, when the surface fraction reaches 70%, efficiency $\varepsilon_{k}$ increases due to connectivity between pixels at the same agglomerate phase.

## 4. Conclusions

The present work presented the relationship between the geometry of a polygonal synthetic agglomerate with respect to effective transport coefficient, considering the percolation effect and surface fraction of both phases. Generation of PSA of 3, 4, 5 sides and circles with random size constructed from mathematical descriptors were obtained to analyze the behavior of each of the configurations in terms of its correlation functions (two-point and line-path correlation functions) acquiring a decay of the S3L image related to the best conduction. Conduction efficiency and local current are affected by the connection between each end of the different configurations (percolation through infinite cluster identifying). Percolation was calculated to establish the necessary elements to ensure the calculation of conduction efficiency in the synthetic image geometry configuration that presents a connection between its edges. PSA samples generated from percolation criteria conclude that the best behavior concerning conduction efficiency is the geometry of three sides (S3L) since it was the only one that percolates in a surface fraction range from 50% to 90%. On the other hand, in the Matrix, the best results are gotten by the SC image, while the lowest is reached by the S3L image. The results computed indicated that the fewer sides the polygon of the microstructure has, there is a higher possibility of reaching percolation, obtaining a better effective electrical conduction, decreasing the variance, and less simulation time. The relationship between percolation calculation and the conduction current is directly dependent on its geometry. The contribution of the work is to present an analysis based on a surface fraction, connectivity, and how its conduction efficiency varies depending on the geometry. The methodology implemented in this work can be extended to experimental design to improve the highest conduction efficiency in membrane electrode assemblies.

## Figures and Tables

**Table 1:** Percolation for agglomerate and matrix phase.

	Percolation (Matrix Phase)									Percolation (Agglomerate Phase)								
PSA	SURFACE FRACTION (%)																	
	10	20	30	40	50	60	70	80	90	10	20	30	40	50	60	70	80	90
S3L	1	1	1	1	1	0	0	0	0	0	0	0	0	1	1	1	1	1
S4L	1	1	1	1	1	0	0	0	0	0	0	0	0	0	0	1	1	1
S5L	1	1	1	1	1	0	0	0	0	0	0	0	0	0	0	1	1	1
SC	1	1	1	1	1	1	0	0	0	0	0	0	0	0	0	1	1	1

## Data Availability

The data that support the findings of this study are available from the corresponding author on reasonable request.

## Nomenclature

Symbol	Description
ETC	Effective Transport Coefficient
MEA	Membrane Electrode Assembly
H2	Hydrogen
FC	Fuel Cells
PEMFC	Proton Exchange Membrane Fuel Cell
CL	Catalytic Layer
RHM	Random Heterogeneous Materials
GDL	Gas Diffuser Layer
PSA	Polygonal Synthetic Agglomerate
FVM	Finite Volume Method
ω	Number of configurations
Ω	Microstructure assembly
W	Number of random series
X	Position of an arbitrary point
r	length of an arbitrary line segment
$T_{\pi }$	Phase function of the phase Π
$\phi_{\pi }$	Surface Fraction of the phase Π
$S_{2,\Pi }(X,R)$	Two-point correlation function
$L_{p,\pi }$	Line-path correlation function
$F_{\left(\Omega ,R\right)}$	Average correlation function
$\chi_{\pi }(x,r)$	Autocovariance function
$\chi_{\pi }^{*}$	Normalized autocovariance function
$L_{\pi }^{*}$	Normalized line-path correlation function
$K_{e}$	Effective conductivity
$K_{M}$	Nominal conductivity
I	Electric current
*J*	Generalized flux
$J_{eff}$	Effective electric flux
*E*	Applied potential
R	Electrical resistance
$\varepsilon_{k}$	Conduction efficiency
*θ*	Angle of the vertex position
*L*	Number of sides of the polygon
*e*	Vertex index
SC	Circular geometry
S3L	3 Sides geometry
S4L	4 Sides geometry
S5L	5 Sides geometry
